# Supplementary figures and images for: Regulating the expression of gene drives is key to increasing their invasive potential and the mitigation of resistance
Source: PLoS Genet. 2021 Jan 29;17(1):e1009321. doi: 10.1371/journal.pgen.1009321 (PMC7886172; doi:10.1371/journal.pgen.1009321)

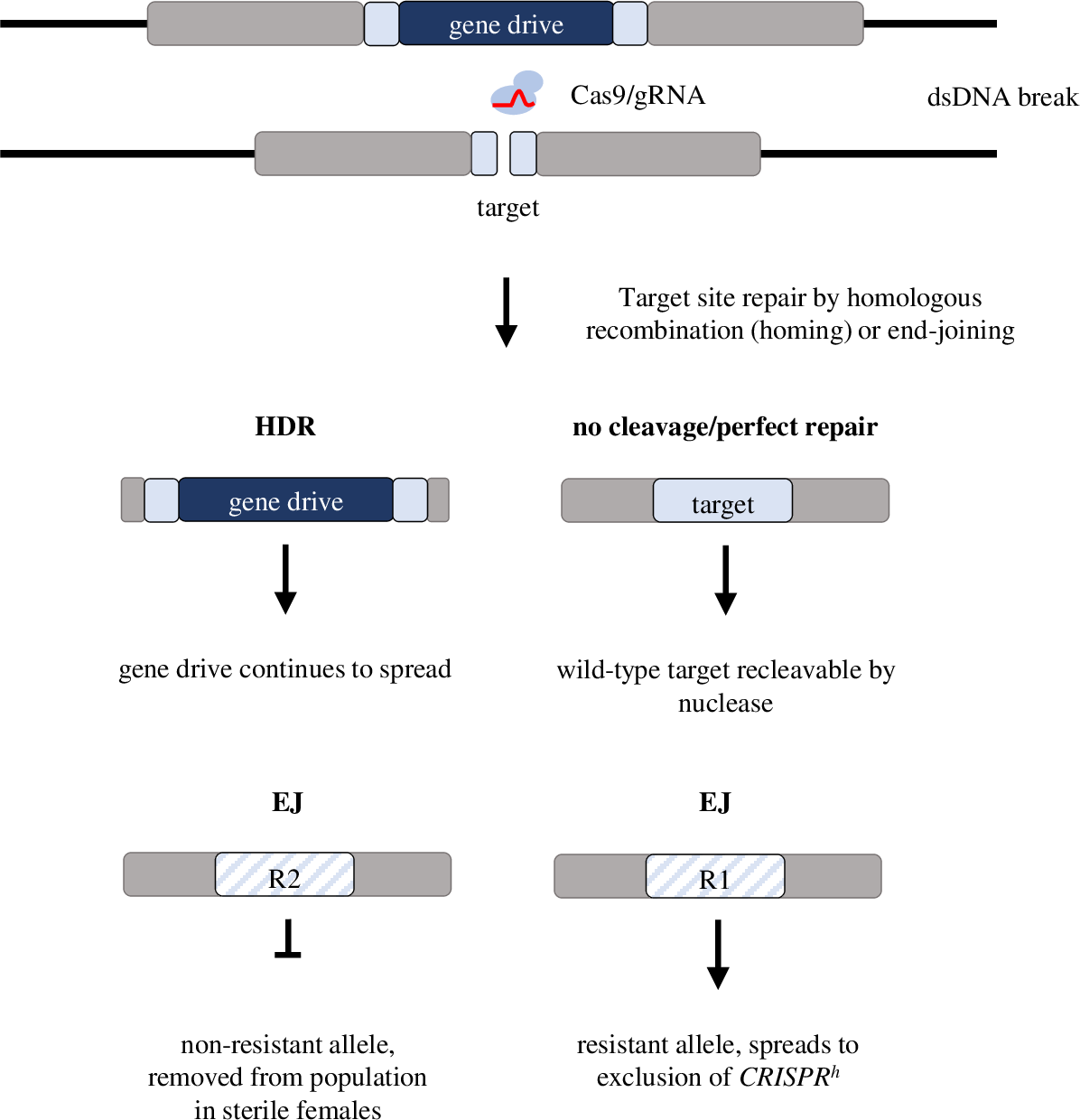

Supplement: S1 Fig — After cleavage by the nuclease, the majority of target sites will be repaired by homology-directed repair (HDR), leading to a copying over of the CRISPRh allele called homing. A small fraction of targets may remain unmodified or may repair perfectly, resulting in a target that can be cleaved upon re-exposure by the nuclease. Chromosomes that are repaired by end-joining may generate a mutant target site that can no longer be cleaved by the nuclease. If the target site is essential (i.e. a female fertility gene), then a mutation that disrupts the function of the target gene, called an r2 mutation, will be selected out of the population. Mutations that re-code a functional target gene, called an r1 mutation, are resistant to the gene drive and will come under strong selection in presence of the drive. (TIF) [file pgen.1009321.s001.tif]

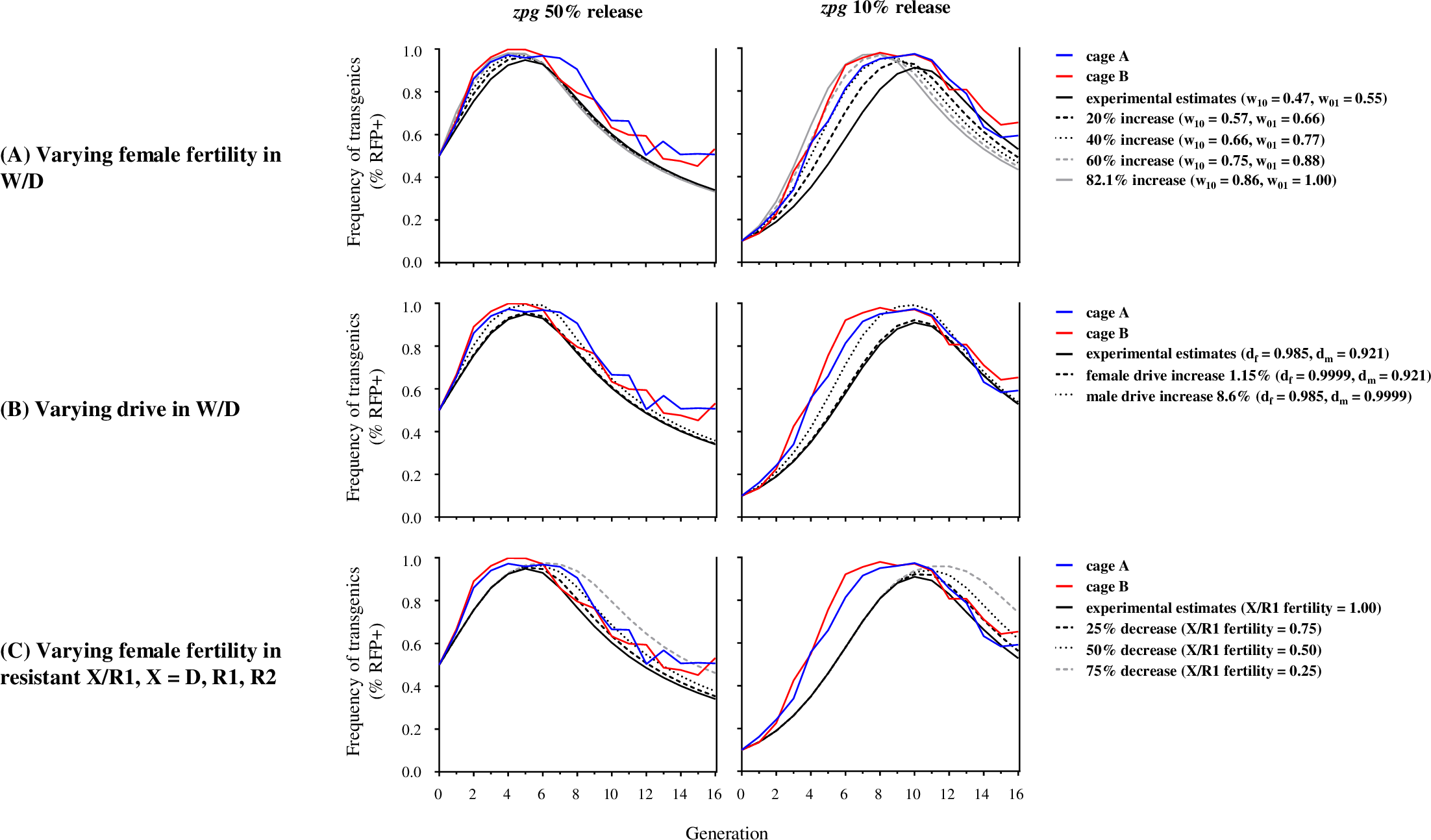

Supplement: S2 Fig — (A) Increasing the fertility of heterozygous zpg-CRISPRh females by up to 80% can accurately predict the experimental data, suggesting the fertility assays underestimate their true fecundity. Note that for (A), W/D female fertilities with maternal/paternal parental effects are varied together, keeping their ratio constant. (B) A small increase in the homing rates of both males and females can have a dramatic effect on the spread of the gene drive during the early generations. By increasing the homing rate estimates of males, similar to that of females, the improved rate of spread better resembles experimental observation. (C) The resilience of the gene drive within the populations increases when the fertility of females with at least one resistant functional allele (r1) is reduced. The reduced output in progeny of these females slows the spread of r1 alleles and thus, allows the drive to spread closer to fixation and to exert a longer lasting and stronger reproductive load on the population. These modelling data suggest that small cage experiments and phenotypic assays provide crude estimates of fertility and drive, and thus both modelling and large-cage testing are needed to better capture and estimate strain performance. (TIF) [file pgen.1009321.s002.tif]

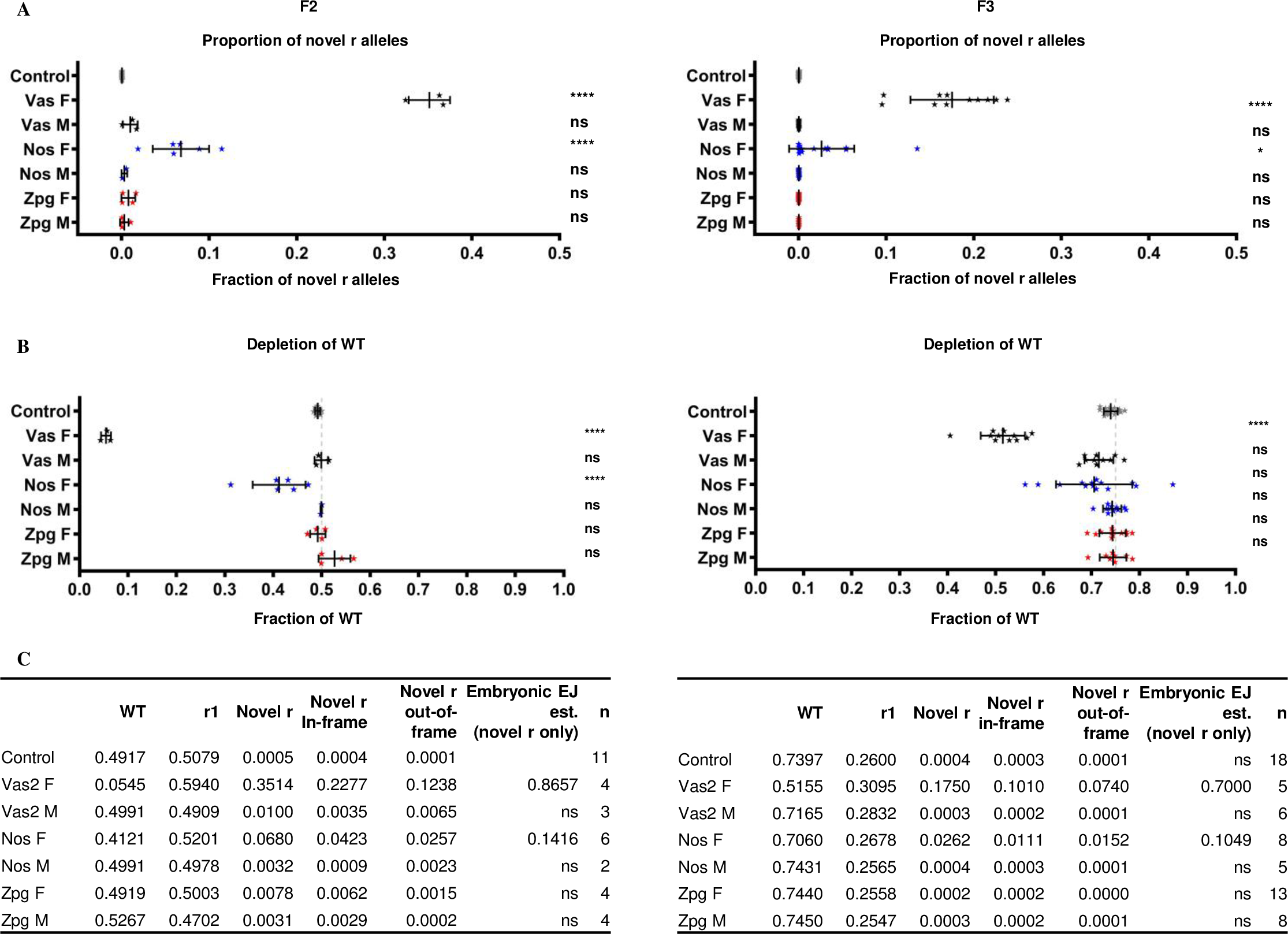

Supplement: S3 Fig — zpg-CRISPRh (‘Zpg’), nos-CRISPRh (‘Nos’)or vas2-CRISPRh (‘Vas’) were crossed to a resistant strain (homozygous for the r1 allele 203-GAGGAG) to generate F1 heterozygotes containing both a gene drive and resistant allele (GD/r1). F1 heterozygote males (M) and females (F) and r1/r1 homozygote females (control) were crossed to wild type and their non-drive F2 progeny analysed by pooled amplicon sequencing across the target site in AGAP007280. (A) Amplicon sequencing results from F2 individuals with genotype WT/r1 (left) and their F3 progeny derived from the F2 crossed to WT (right) are plotted to show the fraction of novel resistant (“R”) alleles (‘Proportion of novel R’, top). (B) Amplicon sequencing results from F2 individuals with genotype WT/r1 (left) and their F3 progeny derived from the F2 crossed to WT (right) are plotted to show the fraction of WT alleles (‘Depletion of WT ‘, middle). Frequencies expected by Mendelian inheritance of the WT allele are indicated (dashed line). Deposited nuclease significantly depleted WT and generated novel R for nos-CRISPRh and vas2-CRISPRh females in the F2, but not zpg-CRISPRh females, or males of any class (Ordinary one-way ANOVA with Dunnett's multiple comparisons test, ****: p < 0.0001, ***: p < 0.001, *: p < 0.05, ns: non-significant). (C) Rates of embryonic EJ were estimated based upon the relative frequencies of novel R alleles in the F2 (left) and F3 (right). F2 estimates were calculated from the frequency of novel R alleles amongst the sum of WT and novel R (left). F3 estimates were calculated by multiplying the frequency of novel R by 4, the dilution factor expected due to additional WT alleles received from the parents (right). A minimum of 128 individuals were sequenced for each condition in the F2 and F3 generations and the number of founder F1 individuals is indicated (n). A summary of this data is shown in Fig 3B and 3C. (TIF) [file pgen.1009321.s003.tif]
