# Supplementary material for: Regulating the expression of gene drives is key to increasing their invasive potential and the mitigation of resistance
Source: PLoS Genet. 2021 Jan 29;17(1):e1009321. doi: 10.1371/journal.pgen.1009321 (PMC7886172; doi:10.1371/journal.pgen.1009321)
Supplement: S1 Text — (DOCX) [file pgen.1009321.s006.docx]

# Hammond et al.

# Supplementary Text

## Supplementary Methods

**Population genetics model**

To model the results of the cage experiments, we use discrete-generation recursion equations for the genotype frequencies, treating males and females separately. $F_{i}(t)$ and $M_{i}\left( t \right)$ denote the frequency of females (or males) of genotype $i=X/Y$in the total female (or male) population. We consider four alleles, $W$ (wildtype), $D$(driver), $R_{1}$(functional resistant) and $R_{2}$(non-functional resistant), and therefore ten basic genotypes. Both resistant alleles cause a change in the target sequence such that it is no longer recognised by the nuclease, but function of the target gene is restored fully or partially in $R_{1}$ alleles and destroyed in $R_{2}$alleles.

**Parental effects.** We consider that further cleavage of the $W$ allele and repair can occur in the embryo if nuclease is present, due to one or both contributing gametes derived from a parent with one or two driver alleles. Previously, embryonic end-joining was modelled as acting immediately in the zygote [1,2]; we consider here that individuals may be mosaics with intermediate phenotypes and therefore we model embryonic activity as causing mosaicism in both the soma, affecting female fitness [3], and also in germline cells, altering gene transmission [4]. No correlation is assumed between the effect on fitness and the effect on gene transmission.

Extending our models [3,4] to include two types of resistance ($R_{1}$and $R_{2}$), we denote mosaic individuals with parental effects (i.e., on fitness and gene transmission rates) as $W/W(a), W/D(a), W/R_{1}(a)$ and $W/R_{2}(a)$, where *a* = 10, 01 or $11$ denotes nuclease from the mother, father or both. Individuals without parental effects are denoted only as X/Y. Gametes are distinguished by whether they carry deposited parental nuclease: W, $R_{1}$,$and R_{2}$ gametes are derived from parents that have no drive allele and therefore carry no deposited nuclease, and gametes $W^{*}$, $D^{*}$, $R_{1}^{*}$,$R_{2}^{*}$are derived from individuals with one or two copies of the drive allele and carry nuclease that is transmitted to the zygote. For example, $W/R_{1}(10)$ mosaic individuals start as zygotes that have either a W* or $R_{1}$* egg from the mother and correspondingly$R_{1}$ or W sperm from the father, and due to the deposited nuclease, the wildtype allele may undergo further cleavage and embryonic end-joining and HDR in the embryo.

**Fitness.** Let $w_{i}\leq1$ represent the fitness of genotype *i* = $X/Y$ relative to $w_{WW}=1$ for the wild-type homozygote. We assume no fitness effects in males. Fitness effects in females are manifested as differences in the relative ability of genotypes to participate in reproduction. We assume the target gene is needed for female participation in reproduction, thus D/D, D/$R_{2}$, and $R_{2}$/$R_{2}$ females do not reproduce, and there is no reduction in fertility in females with only one copy of the gene if no parental effects are present. To model parental effects on fitness (as in [3,4]), genotypes with parental nuclease $W/W(a), W/D(a)$ and $W/R_{2}(a),$ *a* = 10, 01 or $11,$ are assigned an intermediate fitness $w_{WX}^{10}$, $w_{WX}^{01}$, or $w_{WX}^{11}$depending on whether nuclease was derived from a transgenic mother, father, or both (no reduction in fitness of W/$R_{1}$females with parental nuclease, because all cells in the soma have at least one functioning copy of the target gene). We assume that parental effects are the same whether the parent(s) had one or two drive alleles. For simplicity, the same baseline reduced fitness of $w_{10}$, $w_{01}$,$w_{11}$ is assigned to all mosaic genotypes $W/W(a), W/D(a)$ and $W/R_{2}(a$) with maternal, paternal and maternal/paternal effects,*a* = 10, 01 or $11,$ with fitness estimated as the product of mean egg production values and hatching rates relative to wild-type in S1 Table (deterministic model).

**Gene transmission.** We model parental effects on rates of gene transmission from mosaics $W/W(a), W/D(a), W/R_{1}(a)$ and $W/R_{2}(a),$ *a* = 10, 01 or $11,$ by assuming that parentally-derived nuclease can be active in the germline, leading to mosaicism that affects the types and proportions of gametes contributed.

Overall gene transmission from drive individuals includes both greater-than-Mendelian inheritance of the drive allele from germline W/D cells and possible parental effects due to nuclease deposition in the embryo. To incorporate observed measurements of drive into the model, we define overall rates of transmission from W/D individuals, with the proportions of gametes $W^{*}$:$D^{*}$: $R_{1}^{*}$:$R_{2}^{*}$(all carry parental nuclease) from W/D(*a*) individuals depending upon whether the deposited nuclease is from the mother, father, or both ($a = 10, 01 or 11):$

$\left( 1-d_{f}^{a} \right)\left( 1-u_{f}^{a} \right):d_{f}^{a}:\left( 1-d_{f}^{a} \right)u_{f}^{a}\left( 1-\phi\right):\left( 1-d_{f}^{a} \right)u_{f}^{a}\phi$ in females

$\left( 1-d_{m}^{a} \right)\left( 1-u_{m}^{a} \right):d_{m}^{a}:\left( 1-d_{m}^{a} \right)u_{m}^{a}\left( 1-\phi\right):\left( 1-d_{m}^{a} \right)u_{m}^{a}\phi$ in males

Here, $d_{f}^{a}$ and $d_{m}^{a}$are the observed rates of transmission of the driver allele in the two sexes, $u_{f}^{a}$ and $u_{m}^{a}$are the fractions of non-drive gametes that are resistant ($R_{1}^{*}$ and $R_{2}^{*}$alleles), and $\phi$ is the fraction of resistant alleles that are non-functional $R_{2}^{*}$alleles.

In mosaic types $W/W (a),W/R_{1}(a), and W/R_{2}(a)$ with *a* = 10, 01 or $11$ for parental effects due to nuclease deposition from mother, father or both, we define the proportion of wild-type alleles in the germline stem cells that are cleaved and repaired to resistant alleles ($R_{1}$ and $R_{2}$) by end joining as $\delta_{10}$, $\delta_{01}$, $\delta_{11}$with nuclease from mother, father or both, and the proportion repaired by HDR as $\varepsilon_{10}$, $\varepsilon_{01}$, $\varepsilon_{11}$. These parameters are estimated from deposition experiments on W/R1 individuals. S5 Table shows the resulting proportions of different genotypes among germline stem cells (rows) in gonads of mosaic types $W/W (a), W/R_{1}(a), and W/R_{2}(a)$, $a=10, 01 or 11$(columns). Gamete production from $W/W, W/R_{1}$ and $W/R_{2}$ germline stem cells in mosaic individuals is assumed to be Mendelian, since we assume that parental nuclease is no longer active. The resulting proportions of gametes contributed from each type of individual is summarized in S6 Table, along with the fitness for each.

**Recursion equations.** We now consider the gamete contributions from each genotype, including parental effects on fitness and gene transmission. The proportion $e_{k}\left( t \right) {(and s}_{k}\left( t \right))$of type *k* gametes in eggs (and sperm) produced by females (and males) participating in reproduction is given by:

$e_{k}(t)$ =$\sum_{i=1}^{21} A_{i,k}w_{i} F_{i}\left( t \right)/\underline{w}_{f}(t)$ eggs

$s_{k}(t)$ =$\sum_{i=1}^{21} A_{i,k}M_{i}\left( t \right)/\underline{w}_{m}(t)$ sperm

Above, $F_{i}\left( t \right)$ and $M_{i}\left( t \right)$ correspond to the frequencies of female/male individuals of type *i* in the female/male population, where $i$ is summed over the twenty-one individual types (this includes genotypes $F_{XY}\left( t \right)$ and $M_{XY}\left( t \right)$ without parental effects: W/W, W/R_1_, W/ R_2_, D/D, D/R_1_, D/ R_2_ , R_1_/R_1_, R_1_/R_2_, and R_2_/R_2_ and also mosaics $F_{i}^{a}\left( t \right)$and $M_{i}^{a}\left( t \right) o$f genotypes *i* = $W/W, W/D, W/R_{1}$ and $W/R_{2}$distinguished according to parental effect,$a = 10, 01 or 11).$The seven gamete types are distinguished by both the allele that they carry and whether they carry deposited nuclease: $k$ = W, $R_{1}$,$and R_{2}$ (without parental nuclease) and $W^{*}$, $D^{*}$, $R_{1}^{*}$,$R_{2}^{*}$ (with parental nuclease). The coefficients $A_{i,k}$correspond to the proportion of gametes of type $k$ from individuals of type $i$ and are given in S6 Table, with row *i* corresponding to an individual of type *i* and columns to proportions of gametes of type *k*. Above,$w_{i}$ is the fitness of individual of type *i*, where we have assumed all male fitnesses are one, and $\underline{w}_{f}$ and $\underline{w}_{m}$are the average female and male fitnesses:

$\underline{w}_{f}=\sum_{i=1}^{21} w_{i} F_{i}\left( t \right)$ and $\underline{w}_{m}=1$

To model cage experiments, we start with an equal number of males and females and an initial starting frequency of heterozygote drive females and males that inherited the drive from their mothers of${: F}_{WD}^{10}\left( t=0 \right) =M_{WD}^{10}\left( t=0 \right)=0.1 \left( for 10\% release \right)$or $0.5 \left( for 50\% release \right).$ The remaining starting population is wildtype only. Assuming a 50:50 ratio of males and females in progeny, after the starting generation, genotype frequencies of type $i$ in the next generation $(t+1)$ are the same in males and females, $F_{i}\left( t+1 \right)=M_{i}(t+1)$. Both are both given by $G_{i}\left( t+1 \right)$ in the following set of equations in terms of the gamete proportions in the previous generation, assuming random mating:

$$G_{WW}(t+1)=e_{W}(t)s_{W}(t)$$

$$G_{WW}^{10}(t+1)={e_{W}^{*}(t) s}_{W}(t)$$

$$G_{WW}^{01}\left( t+1 \right)=e_{W}(t) s_{W}^{*}(t)$$

$$G_{WW}^{11}\left( t+1 \right)=e_{W}^{*}(t) s_{W}^{*}(t)$$

$$G_{WD}^{10}\left( t+1 \right)=e_{D}^{*}{(t) s}_{W}(t)$$

$$G_{WD}^{01}(t+1)=e_{W}(t) s_{D}^{*}(t)$$

$$G_{WD}^{11}\left( t+1 \right)=e_{W}^{*}{(t)s}_{D}^{*}(t) +e_{D}^{*}{(t) s}_{W}^{*}(t)$$

$$G_{WR_{1}}(t+1)=e_{W}(t)s_{R_{1}}(t)+e_{R_{1}}(t)s_{W}(t)$$

$$G_{WR_{1}}^{10}(t+1)={e_{W}^{*}(t) s}_{R_{1}}(t)+e_{R_{1}(t)}^{*} s_{W}(t)$$

$$G_{WR_{1}}^{01}(t+1)=e_{W}(t) s_{R_{1}}^{*}(t)+e_{R_{1}}(t)s_{W}^{*}(t)$$

$$G_{WR_{1}}^{11}(t+1)=e_{W}^{*}{(t)s}_{R_{1}}^{*}(t)+e_{R_{1}}^{*}(t)s_{W}^{*}(t)$$

$$G_{WR_{2}}(t+1)=e_{W}{(t)s}_{R_{2}}(t)+e_{R_{2}(t)}s_{W}(t)$$

$$G_{WR_{2}}^{10}(t+1)={e_{W}^{*} (t)s}_{R_{2}}(t)+e_{R_{2}}^{*}(t) s_{W}(t)$$

$$G_{WR_{2}}^{01}(t+1)=e_{W}(t) s_{R_{2}}^{*}(t)+e_{R_{2}}(t)s_{W}^{*}(t)$$

$$G_{WR_{2}}^{11}(t+1)=e_{W}^{*}(t)s_{R_{2}}^{*}(t)+e_{R_{2}}^{*}(t)s_{W}^{*}(t)$$

$$G_{DD}\left( t+1 \right)=e_{D}^{*}(t) s_{D}^{*}(t)$$

$$G_{DR_{1}}(t+1)=(e_{R_{1}}(t)+e_{R_{1}}^{*}(t))s_{D}^{*}(t)+e_{D}^{*}(t)(s_{R_{1}}(t)+s_{R_{1}}^{*}(t))$$

$$G_{DR_{2}}(t+1)=\left( e_{R_{2}}(t)+e_{R_{2}}^{*}(t) \right)s_{D}^{*}(t)+e_{D}^{*}(t)\left( s_{R_{2}}(t)+s_{R_{2}}^{*}(t) \right)$$

$$G_{R_{1}R_{1}}(t+1)=(e_{R_{1}}(t)+e_{R_{1}}^{*}(t))(s_{R_{1}}(t)+s_{R_{1}(t)}^{*})$$

$$G_{R_{1}R_{2}}(t+1)=(e_{R_{2}}(t)+e_{R_{2}}^{*}(t))\left( s_{R_{1}}(t)+s_{R_{1}}^{*}(t) \right)+\left( e_{R_{1}}(t)+e_{R_{1}}^{*}(t) \right)\left( s_{R_{2}}(t)+s_{R_{2}}^{*}(t) \right)$$

$$G_{R_{2}R_{2}}(t+1)={(e}_{R_{2}}(t)+e_{R_{2}}^{*}(t))(s_{R_{2}}(t)+s_{R_{2}}^{*}(t))$$

The frequency of transgenic individuals can be compared with experiment (fraction of RFP+ individuals), where here $\left( t \right)$is omitted for brevity:

$f_{RFP+}$ =$F_{WD}^{10}+F_{WD}^{01}+F_{WD}^{11}+F_{DD}+F_{DR_{1}}+F_{DR_{2}}+M_{WD}^{10}+M_{WD}^{01}+M_{WD}^{11}+M_{DD}+M_{DR_{1}}+M_{DR_{2}}$

**Stochastic version.** In the stochastic version of the model described above, random values for probabilistic events are taken from the appropriate multinomial distributions, with probabilities estimated from experiment where applicable (S2 Table). To model the cage experiments, 150 female and 150 male wildtype adults (or 270 females and 270 males for 10% release) along with 150 female and 150 male heterozygotes (or 30 females and 30 males for 10% release) are initially present. Females may fail to mate, or mate once in their life, with a male of a given genotype according to its frequency in the male population, chosen randomly with replacement such that males may mate multiple times. The number of eggs produced from each mated female is randomly chosen by sampling with replacement from experimental values, and the eggs hatch or not with a probability that depends on the mother (S2 Table). To start the next generation, 600 larvae are randomly selected, unless less than 600 larvae have hatched, in which case the smaller amount initiates the next generation, following experiment. The probability of subsequent survival to adulthood is assumed to be equal across genotypes. Assuming very large population sizes gives results for the genotype frequencies that are indistinguishable from the deterministic model. For the deterministic egg count, we use the large population limit of the stochastic model.

All calculations are carried out using Wolfram Mathematica [5].

**Supplementary References**

[1] Papathanos, P. A., Windbichler, N., Menichelli, M., Burt, A. and Crisanti, A. The vasa regulatory region mediates germline expression and maternal transmission of proteins in the malaria mosquito *Anopheles gambiae:* a versatile tool for genetic control strategies. *BMC Mol Biol* **10**, 65, (2009).

[2] Hammond, A.M. et al. The creation and selection of mutations resistant to a gene drive over multiple generations in the malaria mosquito. *PLoS Genet* **13**, e1007039 (2017).

[3] Kyrou K., Hammond A.M., Galizi R., Kranjc N., Burt A., Beaghton A.K., et al. 2018 A CRISPR-Cas9 gene drive targeting doublesex causes complete population suppression in caged Anopheles gambiae mosquitoes. *Nat Biotechnol* **36**(11), 1062-1066. (doi:10.1038/nbt.4245)

[4] Beaghton A.K., Hammond A., Nolan T., Crisanti A., Burt A. 2019 Gene drive for population genetic control: non-functional resistance and parental effects. *Proc Biol Sci* **286** (1914), 20191586. (doi:10.1098/rspb.2019.1586)

[5] Wolfram Research, Inc., 2017 Mathematica 11.2, Champaign, IL.
