# Supplementary material for: Regulating the expression of gene drives is key to increasing their invasive potential and the mitigation of resistance
Source: PLoS Genet. 2021 Jan 29;17(1):e1009321. doi: 10.1371/journal.pgen.1009321 (PMC7886172; doi:10.1371/journal.pgen.1009321)
Supplement: S1 Table — (DOCX) [file pgen.1009321.s007.docx]

## S1 Table

**S1 Table | Model parameters for zpg-CRISPR^h^**

| **Parameter** | **Symbol** | **Estimate** | **Method of estimation** |
| --- | --- | --- | --- |
| Average fertility  W/D females from female | $w_{10}$ | 0.471^ǂ^ | Phenotype assay (mean larvae count, relative to WT value) |
| Average fertility  W/D females from male | $w_{01}$ | 0.549^ǂ^ | Phenotype assay (mean larvae count, relative to WT value) |
| Drive in  W/D females from female | $d_{f}^{10}$ | 0.985 | Phenotype assay |
| Drive in  W/D females from male | $d_{f}^{01}$ | 0.985 | Phenotype assay |
| Drive in  W/D males from female | $d_{m}^{10}$ | 0.921 | Phenotype assay |
| Drive in  W/D males from male | $d_{m}^{01}$ | 0.921 | Phenotype assay |
| EJ parameter in W/D (fraction non-drive alleles that are resistant*)* | *u^*^* | 0.392 | Sequencing non-drive progeny of zpg-CRISPR^h^ heterozygotes |
| Fraction resistant alleles that are non-functional | *φ* | 0.378 | Single generation resistance assay using zpg-CRISPR^h^ |
| Embryonic HDR in  W/R1 from female | $\epsilon_{10}$ | 0.0 | Deposition induced EJ and HDR |
| Embryonic HDR in  W/R1 from male | $\epsilon_{01}$ | 0.0 | Deposition induced EJ and HDR |
| Embryonic EJ in  W/R1 from female | $\delta_{10}$ | 0.0 | Deposition induced EJ and HDR |
| Embryonic EJ in  W/R1 from male | $\delta_{01}$ | 0.0 | Deposition induced EJ and HDR |
| Initial frequency of heterozygous drive individuals (that inherited drive from a female parent) | $F_{WD}^{10} ,M_{WD}^{10}$ | $0.1 \left( 10\% release \right)$  $0.5 (50\% release)$ | Following cage experiment |

ǂ Deterministic model

*^*^*For comparison to experiment, it is assumed that this parameter is the same in females and males and with maternal or paternal effect, $u_{F}^{10}=u_{F}^{01}=u_{M}^{10}=u_{M}^{01}=u_{M}^{11}=u_{M}^{11}=u$.

**S1 Table | Model parameters for zpg-CRISPR^h^.** For combined maternal and paternal effects (nuclease from both parents), the minimum of observed values for maternal or paternal effect is used for the fitness ($w_{11}$) and drive ($d_{f}^{11}$and $d_{m}^{01}$); for embryonic HDR and EJ parameters $\epsilon_{11}$ and $\delta_{11}$, we use the maximum observed value of each from maternal or paternal effect. We assume that parental effects on fitness and embryonic HDR and EJ parameters for non-drive (W/W, W/R_2_) females with nuclease from one or both parents are the same as observed values for drive heterozygote (W/D) females with parental effects.
