# Supplementary material for: Regulating the expression of gene drives is key to increasing their invasive potential and the mitigation of resistance
Source: PLoS Genet. 2021 Jan 29;17(1):e1009321. doi: 10.1371/journal.pgen.1009321 (PMC7886172; doi:10.1371/journal.pgen.1009321)
Supplement: S2 Table — (DOCX) [file pgen.1009321.s008.docx]

## S2 Table

**S2 Table | Additional parameters for stochastic model for zpg-CRISPR^h^**

| **Parameter** | **Estimate** | **Method of estimation** |
| --- | --- | --- |
| Mating probability | 0.85 | Hammond et al. 2017 |
| Egg count  wildtype | {0, 0, 61, 63, 66, 77, 77, 92, 93, 99, 107, 108, 109, 109, 111, 113, 115, 116, 116, 118, 119, 120, 120, 121, 121, 124, 126, 127, 127, 127, 128, 129, 130, 130, 132, 134, 135, 136, 140, 144, 151, 153, 161, 162, 171} (Mean 113.7) | Phenotype assay |
| Egg count  W/D female from female | {0, 0, 0, 0, 17, 17, 20, 20, 39, 42, 44, 45, 47, 47, 50, 59, 60, 60, 61, 61, 67, 67, 77, 80, 88, 88, 94, 97, 104, 107, 110, 111, 117, 119, 122, 148, 154, 155} (Mean 68.3) | Phenotype assay |
| Egg count  W/D female from male | {0, 12, 36, 41, 42, 43, 51, 57, 61, 61, 64, 66, 67, 68, 70, 71, 76, 81, 83, 87, 88, 89, 91, 93, 93, 94, 102, 105, 106, 110, 115, 115, 116, 127, 128, 135, 182} (Mean 81.8) | Phenotype assay |
| Hatching rate,  wildtype  (no parental nuclease) | 0.954 | Phenotype assay (mean larvae count divided by mean egg count) |
| Hatching rate,  W/D female from female | 0.748 | Phenotype assay (mean larvae count divided by mean egg count) |
| Hatching rate,  W/D female from male | 0.729 | Phenotype assay (mean larvae count divided by mean egg count) |
| Probability of emergence from pupa (survival from larva) | 0.73 | Average over all gens. and cage experiments |
| Initial population (zero generation) | 50% (10%) release: 150 (270) female and 150(270) male WT adults, 150 (30) female and 150 (30) male heterozygous zpg-CRISPR^h^ that inherited drive from a female parent. | Following cage experiment |

**S2 Table | Additional parameters for stochastic model for zpg-CRISPR^h^**. We assume that parental effects on fitness (egg production and hatching rates) for non-drive (W/W, W/R_2_) females with nuclease from one or both parents are the same as observed values for female drive heterozygote (W/D) females with parental effects.
