# Supplementary material for: Regulating the expression of gene drives is key to increasing their invasive potential and the mitigation of resistance
Source: PLoS Genet. 2021 Jan 29;17(1):e1009321. doi: 10.1371/journal.pgen.1009321 (PMC7886172; doi:10.1371/journal.pgen.1009321)
Supplement: S3 Table — (DOCX) [file pgen.1009321.s009.docx]

## S3 Table

| **S3 *Table* \| Primers used in this study to assemble the vectors** | |
| --- | --- |
| nos-pr-CRISPR-F | GCTCGAATTAACCATTGTGGACCGGTGTGAACTTCCATGGAATTACGT |
| nos-pr-CRISPR-R | TCGTGGTCCTTATAGTCCATCTCGAGCTTGCTTTCTAGAACAAAAGGATC |
| nos-ter-CRISPR-F | GCCGGCCAGGCAAAAAAGAAAAAGTAATTAATTAAGACAGAGTCGTTCGTTCATT |
| nos-ter-CRISPR-r | TCAACCCTTCAAGCGCACGCATACAAAGGCGCGCCGTAATTAGTGTTCATTTTAG |
| zpg-pr-CRISPR-F | GCTCGAATTAACCATTGTGGACCGGTCAGCGCTGGCGGTGGGGA |
| zpg-pr-CRISPR-R | TCGTGGTCCTTATAGTCCATCTCGAGCTCGATGCTGTATTTGTTGT |
| zpg-ter-CRISPR-F | AGGCAAAAAAGAAAAAGTAATTAATTAAGAGGACGGCGAGAAGTAATCAT |
| zpg-ter-CRISPR-R | TTCAAGCGCACGCATACAAAGGCGCGCCTCGCATAATGAACGAACCAAAGG |
| exu-pr-CRISPR-F | GCTCGAATTAACCATTGTGGACCGGTGGAAGGTGATTGCGATTCCATGT |
| exu-pr-CRISPR-R | TCGTGGTCCTTATAGTCCATCTCGAGTTTGTACAAGCTACACAAGAGAAGG |
| exu-ter-CRISPR-F | AGGCAAAAAAGAAAAAGTAATTAATTAAGCGTGAGCCGGAGAAAGC |
| exu-ter-CRISPR-R | TTCAAGCGCACGCATACAAAGGCGCGCCACTGCTACTGTGCAACACATC |

**S3 Table.** Primers used to amplify *nos*, *zpg* and *exu* promoter (pr) and terminator (ter) sequences. Underlined are the Gibson adaptors used to clone promoter and terminator fragments into the CRISPR^h^ vector.
