# Supplementary material for: Regulating the expression of gene drives is key to increasing their invasive potential and the mitigation of resistance
Source: PLoS Genet. 2021 Jan 29;17(1):e1009321. doi: 10.1371/journal.pgen.1009321 (PMC7886172; doi:10.1371/journal.pgen.1009321)
Supplement: S4 Table — (DOCX) [file pgen.1009321.s010.docx]

## S4 Table

| **S4 Table – Promoter and terminator sequences** | |
| --- | --- |
| Zpg promoter (1074bp)  930bp upstream of AGAP006241 + 144bp putative 5’ UTR (underlined) | cagcgctggcggtggggacagctccggctgtggctgttcttgCgagtcCtcttcctgcggcacatccctctcgtcgaccagttcagtttgctgagcgtaagcctgctgctgttcgtcctgcatcatcgggaccatttgtaTgggccatccgccaccaccaccatcaccaccgccgtccatttctaggggcatacccatcagcatctccgcgggcgccattggcggtggtgccaaggtgccattcgtttgttgctgaaagcaaaagaaagcaaattagtgttgtttctgctgcacacgataAttttcgtttcttgccgctagacacaaacaacactgcatctggagggagaaatttgacgcctagctgtataacttacctcaaagttattgtccatcgtggtataatggacctaccgagcccggttacactacacaaagcaagattatgcgacaaaatcacagcgaaaactagtaattttcatctatcgaaagcggccgagcagagagttgtttggtattgcaacttgacattctgctgCgggataaaccgcgacgggctaccatggcgcacctgtcagatggctgtcaaatttggcccggtttgcgatatggagtgggtgaaattatatcccactcgctgatcgtgaaaatagacacctgaaaacaataattgttgtgttaattttacattttgaagaacagcacaagttttgctgacaatatttaattacgtttcgttatcaacggcacggaaagattatctcgctgattatccctctcgctctctctgtctatcatgtcctggtcgttctcgcgtcaccccggataatcgagagacgccatttttaatttgaactactacaccgacaagcatgccgtgagctctttcaagttcttctgtccgaccaaagaaacagagaataccgcccggacagtgcccggagtgatcgatccatagaaaatcgcccatcatgtgccactgaGgcgaaccggcgtagcttgttccgaatttccaagtgcttccccgtaacatccgcatataacaaAcagcccaacaacaaatacagcatcgag |
| Zpg terminator (1037bp)  (20bp putative 3’ UTR + 1017bp upstream of AGAP006241) | Gaggacggcgagaagtaatcatatgtccgcattttgcgcaaaccaggcgcttagacaatttgcgcgtaagcacattcgaaatgtgaaaagctgaaagcagtggtttcgccagcccgagttcagcgaaacggattccttccaagtgtttgcattcctggcggagtgttcctcccaaaatgcactcaccctgcgtgcagtgccaaatcgtgagtttcctaattttttcatattgtttattacctaccaactaaagttgttgttatatattgcgttttacgtacgacaaataagttcgtattcagaaatatttgcgataagagagaactcatttgcgatgaatctcattgtatttagctaagtgccttgataagtaagcggaacagcaggaatatgacactccttgggaaatacatgtaagcgtctgtaattagatatatatacacgcaaccaaatggtccatggttgatttaagcactgcctgttgtcgaacattgctataagcaaaataaagaagcattcattaatctaaaatttcttcaaagtgacttcaatgatgatctctaggctatagtgaaagctgaaagcttatttgacaatgcaagggaaagtgacgcacgtgcgtcgtatgggaccgcgcgcatctattctctcagctaattcccctaatcattagtaattgacggcacgatttctgcttcttacttccttttactttggagcttttcatcaataaaaccagtaccatggccgtacgctcaacggaaaagcattcaaaaaaacccgcgttcctcgtgtgatttgtgggtgagtggcgccatctattagagaatagctgtactacatctcgtggacgaaggggtcagagaagttgaaagagagcttgatcgactgctatccaagctaggcgaggaagggagatcgctagagcaaaagaaaaaaaataagcaaatatctttttttataacaaatcgacgttagcgaaatatgtttgaatcgatttaacggttagaattccctttggttcgttcattatgcga |
| Nos promoter (2092)  1791bp upstream of AGAP006098 + 202bp putative 5’ UTR (underlined) + 91bp intron 1 (bold) + 8bp 5’UTR | gtgaacttccatggaattacgtgctttttcggaatggagttgggctggtgaaaaacacctatcagcaccgcacttttcccccggcatttcaggttatacgcagagacagagactaaatattcacccattcatcacgcactaacttcgcaatagattgatattccaaaactttcttcacctttgccgagttggattctggattctgagactgtaaaaagtcgtacgagctatcatagggtgtaaaacggaaaacaaacaaacgtttaatggactgctccaactgtaatcgcttcacgcaaacaaacacacacgcgctgggagcgttcctggcgtcacctttgcacgatgaaaactgtagcaaaactcgcacgaccgaaggctctccgtccctgctggtgtgtgtttttttcttttctgcagcaaaattagaaaacatcatcatttgacgaaaacgtcaactgcgcgagcagagtgaccagaaataccgatgtatctgtatagtagaacgtcggttatccgggggcggattaaccgtgcgcacaaccagttttttgtgcagctttgtagtgtctagtggtattttcgaaattcatttttgttcattaacagttgttaaacctatagttattgattaaaataatattctactaacgattaaccgatggattcaaagtgaataaattatgaaactagtgatttttttaaatttttatatgaatttgacatttcttggaccattatcatcttggtctcgagctgcccgaataatcgacgttctactgtattcctaccgattttttatatgcctaccgacacacaggtgggccccctaaaactaccgatttttaatttatcctaccgaaaatcacagattgtttcataatacagaccaaaaagtcatgtaaccatttcccaaatcacttaatgtattaaactccatatggaaatcgctagcaaccagaaccagaagttcaacagagacaaccaatttccgtgtatgtacttcatgagatgagattggacgcgctggtaaaattttatatgggatttgacagataatgtaaggcgtgcgatttttttcatacgatggaatcaattcaagagtcaattgtgcaggatttatagaaacaatctcttatttatgttttgttatcgttacagttacagccctgtcctaagcggccgcgtgaaggcccaaaaaaaagggagtccccaacgctcagtagcaaatgtgcttctctatcattcgttgggttagaaaagcctcatgtgacttctatgaacaaaatctaaactatctcctttaaatagagaatggatgtattttttcgtgccactgaactttcgttgggaagattagatacctctccctccccccccctccctttcaacacttcaaaacctaccgaaaactaccgatacaatttgatgtacctaccgaagaccgccaaaataatctggccacactggctagatctgatgttttgaaacatcgccaaattttactaaataatgcacttgcgcgttggtgaagctgcacttaaacagattagttgaattacgctttctgaaatgtttttattaaacacttgttttttttaatacttcaatttaaagctacttcttggaatgataattctacccaaaaccaaaaccactttacaaagagtgtgtggttggtgatcgcgccggctactgcgacctgtggtcatcgctcatctcacgcacacatacgcacacatctgtcatttgaaaagctgcacacaatcgtgtgttgtgcaaaaaaccgttcgcgcacaaacagttcgcacatgtttgcaagccgtgcagcaaagggcttttgatggtgatccgcagtgtttggtcagctttttaatgtgttttcgcttaatcgcttttgtttgtgtaatgttttgtcggaataatttttatgcgtcgttacaaatgaaatgtacaatcctgcgatgctagtgtaaaacattgctaattcccg**gtaagaacgttcattacgctcggatatcatcttacgaagcgTGTGTATGTGCGCTAGTACATTGACCTTTAAAGTgatccttttgttctag**aaagcaag |
| Nos terminator (601bp)  (150bp putative 3’ UTR + 451bp downstream of AGAP006098) | gacagagtcgttcgttcattccttttttattactttacaacacatccaaagctctgtgagcttcaagcaacaggtagtagctgacatcggaactggtgggcaagaaaggcttTcagcaaatatgtttCAggctgctcggagaatgttgaagatatatttatttaggaaaagtggaactttatgcaggatgaataattttgccatcgaatcaaatagcgtaagtaggtagagtgaaaaatTgatcttaaaaggatgatttccacgttcgaacattacacattaaggatggtatccatacatacgaatgcggtttaaattcaatatttaccttgaagcagGtgttcgtatcatttcctccttagcatctttatgtctaAacttctttCaaTgacaacatttatCgattttttgatacaacgaatcattttctatgaatcaatcaCttgatgcCttgattaaataaattgcgaagaaatatttaacaacaatcgattctaaatgtgttgttagaagAtacaaccaaaaaacctttaatAtcttggagcgaatgttcaaagatattgTttagcctctctcttcggctaaaatgaacactaattac |
| Exu promoter (849bp)  654bp upstream of AGAP007365 + 192bp putative 5’ UTR (underlined) + 83bp intron 1 (bold) + 10bp putative 5’ UTR (underlined) | ggaaggtgattgcgattccatgttgatgccaatatatgatgattttgttgcatattaatagttgttgttatgttttattcaaatttcaaagataatttactttacattacagttagtgagcatattatctactacataaacacatagatCaaactggtttacataaattcaaaaagtttgGattaaAatcgcagcaattggttatgaaaaaatatgtgCAtaacgtaaatatcaagtaaatttttgcattgcatatttatagaCtcctgttacaatttcggaaaaatgaaaaatgttaattaatcaaagaagaaaaaacaaagAaattaaatcattaggtAgcacaaccacaagtacatatttttatggcatgaatattccTctacactaacatattttatagcaattctattgatcgccttaGtatagcGgaattaccagaacggcactatagttgtctctgtttggcacacgcaatcatttttcatcccagggttgccatagcagtttggcgacggtcacgtagcatgcgaaggatttcgTtcgcacaggatcacttttattctaacgtttgaagaagGcacatctcagtgcaagcgctctggaagctgcttttaccgaacgaactaacttttcaagtaacctcaaaaacttgtctctaacgacaccacgtgctatccgcgagttTcatttcccgtgcaaagttccccgatttagctatcattcgtgaacatttcgtagtgcctctaccctcag**gtaagaccattcgaGgtttaccaagttttgtgcaaagaaCGTGCacagtaattttCgttctggtgaaaccttctcttgtgtag**cttgtacaaa |
| Exu terminator (1173bp)  (627bp putative 3’ UTR + 546bp downstream of AGAP007365) | gcgtgagccggagaaagcttgcgggacattttaacggtagatgtctgatgattgttgcatagatgcagtagatgcaggaacatacccgattcaaagaacatctttttacggataatggcaaggaatacgaaacatttttaaacacgctcaaatagatttaaggatcatcacgatagaagtagccaactcattcggtatagttgtcctcgtattttagaatcaggtagaagcgctgcttcagcagcaaaatgtcttagctcaggatcatagggaaggatggccgttgaacccgttgaaagtatgcgcgcactttctggggtaatttccaaggcgtcatccggcccacttttagtcacccagaagctaggaccgtttgcttgcatttgtgtggtgcagaaccccattttaatattgtgtaaattattttcactacgtagcaatcaataccaatgtgaggctgcaaaacgtctcttttagctgtggtgggttgtagcagctcagaaatgaaatcaacgatttatgttatgattgtaattgatggaaggtgtacaacacggaagggccagacctctggaaggcgatggcggttccatgttgatgccaatatatgatgattttgttgcattgaatatttgtttgcttgttctatttgattagtgggttgaatttggaaagaaatgtacgatattcggatggagttatgggtgtacaacagtagtgtctgtagttagtgtactaattgtgattaagattttgaattttatttccctttttgagacagaacatttatcgcaggctggacctggctatccagctaccctcgcgtgtcttttgtaccaattgggcattaactatcctgtctagaatgagcttgcttgtgtgtctgtggctcaatgtacgcgctttggggaggaagaaaactgttggtacaattatactactcatgcatctagtatcatgtaaatacactcaaaacatcaatcaatccatcaatagtagttactccttcattagtgcctaggaacgcactgcactaccgatgcagcgccggagaaaacatgtatccttgcgtttgtgtcactagtcctatatatacccaaaactgcccccaacaatcgtgctcaaagtacggttaatacggggcagcggggagatgtgttgcacagtagcagt |

Note: Green annotation indicates differences between the online reference sequence (PEST strain) and our lab colony (G3 strain).
