# Supplementary material for: Regulating the expression of gene drives is key to increasing their invasive potential and the mitigation of resistance
Source: PLoS Genet. 2021 Jan 29;17(1):e1009321. doi: 10.1371/journal.pgen.1009321 (PMC7886172; doi:10.1371/journal.pgen.1009321)
Supplement: S5 Table — (DOCX) [file pgen.1009321.s011.docx]

## S5 Table

**S5 Table | Proportions of germline cells in W/Y (Y = W,** $R_{1}$**,** $R_{2}$**) with parental effects**

| Germline |  | | Mosaic genotype |  | |
| --- | --- | --- | --- | --- | --- |
| stem cell | $G_{WW}^{a}$ | $G_{WR_{1}}^{a}$ | | | $G_{WR_{2}}^{a}$ |
| $W/W$ | ${(1-\delta_{G}^{a})}^{2}$ | $0$ | | | $0$ |
| $W/R_{1}$ | $2(1-\phi_{e})(1-\delta_{G}^{a})\delta_{G}^{a}$ | $1-\varepsilon_{G}^{a}-\delta_{G}^{a}$ | | | $0$ |
| $W{/R}_{2}$ | $2\phi_{e}(1-\delta_{G}^{a})\delta_{G}^{a}$ | $0$ | | | $1-\varepsilon_{G}^{a}-\delta_{G}^{a}$ |
| $R_{1}/R_{1}$ | ${(1-\phi_{e})}^{2}{(\delta_{G}^{a})}^{2}$ | $\varepsilon_{G}^{a}+(1-\phi_{e})\delta_{G}^{a}$ | | | $0$ |
| $R_{1}/R_{2}$ | $2(1-\phi_{e})\phi_{e}{(\delta_{G}^{a})}^{2}$ | $\phi_{e}\delta_{G}^{a}$ | | | $(1-\phi_{e})\delta_{G}^{a}$ |
| $R_{2}/R_{2}$ | ${(\phi_{e})}^{2}{(\delta_{G}^{a})}^{2}$ | $0$ | | | $\varepsilon_{G}^{a}+\phi_{e}\delta_{G}^{a}$ |

**S5 Table.** For the model of parental effects, the proportions of germline stem cells of different genotypes in female or male ($G_{i}^{a}=\{F_{i}^{a},M_{i}^{a}\}$ mosaics (derived from zygotes of W/W, W/R_1_ and W/R_2_) where the nuclease is deposited from a mother, father or both ($a=\{10, 01, 11\})$.
