# Supplementary material for: Regulating the expression of gene drives is key to increasing their invasive potential and the mitigation of resistance
Source: PLoS Genet. 2021 Jan 29;17(1):e1009321. doi: 10.1371/journal.pgen.1009321 (PMC7886172; doi:10.1371/journal.pgen.1009321)
Supplement: S6 Table — (DOCX) [file pgen.1009321.s012.docx]

## S6 Table **Genotype fitnesses and the proportion of each type of gamete produced by them**

| Genotype ǂ | Fitness |  |  | Gametes produced |  |  | |  | |  |
| --- | --- | --- | --- | --- | --- | --- | --- | --- | --- | --- |
|  |  | W | R_1_ | R_2_ | W* | D* | R_1_* | | R_2_ * | |
| W/W | $1$ | $1$ | $0$ | $0$ | $0$ | $0$ | $0$ | | $0$ | |
| W/W (10) | $w_{10}$ | ${1-\delta}_{G}^{10}$ | $\delta_{G}^{10}\left( 1-\phi_{e} \right)$ | $\delta_{G}^{10}\phi_{e}$ | $0$ | $0$ | $0$ | | $0$ | |
| W/W (01) | $w_{01}$ | ${1-\delta}_{G}^{01}$ | $\delta_{G}^{01}\left( 1-\phi_{e} \right)$ | $\delta_{G}^{01}\phi_{e}$ | $0$ | $0$ | $0$ | | $0$ | |
| W/W (11) | $w_{11}$ | ${1-\delta}_{G}^{11}$ | $\delta_{G}^{11}\left( 1-\phi_{e} \right)$ | $\delta_{G}^{11}\phi_{e}$ | $0$ | $0$ | $0$ | | $0$ | |
| W/D (10) | $w_{10}$ | $0$ | $0$ | $0$ | $(1-d_{G}^{10})(1-u_{G}^{10})$ | $d_{G}^{10}$ | $\left( 1-d_{G}^{10} \right)u_{G}^{10}(1-\phi)$ | | $\left( 1-d_{G}^{10} \right)u_{G}^{10}\phi$ | |
| W/D (01) | $w_{01}$ | $0$ | $0$ | $0$ | $(1-d_{G}^{01})(1-u_{G}^{01})$ | $d_{G}^{10}$ | $\left( 1-d_{G}^{01} \right)u_{G}^{01}(1-\phi)$ | | $\left( 1-d_{G}^{01} \right)u_{G}^{01}\phi$ | |
| W/D (11) | $w_{11}$ | $0$ | $0$ | $0$ | $(1-d_{G}^{11})(1-u_{G}^{11})$ | $d_{G}^{10}$ | $\left( 1-d_{G}^{11} \right)u_{G}^{11}(1-\phi)$ | | $\left( 1-d_{G}^{11} \right)u_{G}^{11}\phi$ | |
| W/R_1_ | 1 | $1/2$ | $1/2$ | $0$ | $0$ | $0$ | $0$ | | $0$ | |
| W/ R_1_ (10) | 1 | $\left( {1-\epsilon_{G}^{10}-\delta}_{G}^{10} \right)/2$ | $\left( {1+\epsilon_{G}^{10}+\delta}_{G}^{10}\left( 1-\phi_{e} \right) \right)/2$ | $\delta_{G}^{10}\phi_{e}/2$ | $0$ | $0$ | $0$ | | $0$ | |
| W/ R_1_ (01) | 1 | $\left( {1-\epsilon_{G}^{01}-\delta}_{G}^{01} \right)/2$ | $\left( {1+\epsilon_{G}^{01}+\delta}_{G}^{01}\left( 1-\phi_{e} \right) \right)/2$ | $\delta_{G}^{01}\phi_{e}/2$ | $0$ | $0$ | $0$ | | $0$ | |
| W/ R_1_ (11) | 1 | $\left( {1-\epsilon_{G}^{11}-\delta}_{G}^{11} \right)/2$ | $\left( {1+\epsilon_{G}^{11}+\delta}_{G}^{11}\left( 1-\phi_{e} \right) \right)/2$ | $\delta_{G}^{11}\phi_{e}/2$ | $0$ | $0$ | $0$ | | $0$ | |
| W/R_2_ | 1 | $1/2$ | $0$ | $1/2$ | $0$ | $0$ | $0$ | | $0$ | |
| W/R_2_ (10) | $w_{10}$ | $\left( {1-\epsilon_{G}^{10}-\delta}_{G}^{10} \right)/2$ | $\delta_{G}^{10}\left( 1-\phi_{e} \right)/2$ | $\left( {1+\epsilon_{G}^{10}+\delta}_{G}^{10}\phi_{e} \right)/2$ | $0$ | $0$ | $0$ | | $0$ | |
| W/R_2_ (01) | $w_{01}$ | $\left( {1-\epsilon_{G}^{01}-\delta}_{G}^{01} \right)/2$ | $\delta_{G}^{01}\left( 1-\phi_{e} \right)/2$ | $\left( {1+\epsilon_{G}^{01}+\delta}_{G}^{01}\phi_{e} \right)/2$ | $0$ | $0$ | $0$ | | $0$ | |
| W/R_2_ (11) | $w_{11}$ | $\left( {1-\epsilon_{G}^{11}-\delta}_{G}^{11} \right)/2$ | $\delta_{G}^{11}\left( 1-\phi_{e} \right)/2$ | $\left( {1+\epsilon_{G}^{11}+\delta}_{G}^{11}\phi_{e} \right)/2$ | $0$ | $0$ | $0$ | | $0$ | |
| D/D | 0 | $0$ | $0$ | $0$ | $0$ | $1$ | $0$ | | $0$ | |
| D/ R_1_ | 1 | $0$ | $0$ | $0$ | $0$ | $1/2$ | $1/2$ | | $0$ | |
| D/ R_2_ | 0 | $0$ | $0$ | $0$ | $0$ | $1/2$ | $0$ | | $1/2$ | |
| R_1_/R_1_ | 1 | $0$ | $1$ | $0$ | $0$ | $0$ | $0$ | | $0$ | |
| R_1_/R_2_ | 1 | $0$ | $1/2$ | $1/2$ | $0$ | $0$ | $0$ | | $0$ | |
| R_2_/R_2_ | 0 | $0$ | $0$ | $1$ | $0$ | $0$ | $0$ | | $0$ | |

ǂ(10),(01), or (11) denotes a mosaic type with effects of deposited parental nuclease from mother, father or both.

**S6 Table.** The fitnesses of the 21 types (10 basic genotypes differentiated according to parental effect) and the proportion of each type of gamete produced by them. *G*={*F, M*}, where *F* denotes females and eggs produced; *M* denotes males and sperm produced.
