# Supplementary material for: Regulating the expression of gene drives is key to increasing their invasive potential and the mitigation of resistance
Source: PLoS Genet. 2021 Jan 29;17(1):e1009321. doi: 10.1371/journal.pgen.1009321 (PMC7886172; doi:10.1371/journal.pgen.1009321)
Supplement: Attachment — Submitted filename: response to reviewers PLoS Gen (1).docx [file pgen.1009321.s013.docx]

Dear Professor Edwards,

Thank you very much for handling our manuscript. We are particularly grateful to each of the reviewer’s for the detailed and thorough analysis of our manuscript and for their warm appraisal of how it advances the field. It is very rewarding to receive such constructive comments from one's peers and all the more so at the moment. That goes from the corresponding author to the most junior on the manuscript. We hope that we have responded to these comments satisfactorily. Without doubt this process has greatly improved the clarity of the article and its presentation. We have responded to each comment in line below (**in bold**) and have submitted the revised version of the manuscript attached to this letter

Yours sincerely,

Tony Nolan

**RESPONSES**

Reviewer #1

*This group has addressed many of these issues by targeting a female-splice form of the doublesex (dsx) gene, which apparently cannot (thus far) mutate to a functional but uncleavable (resistant) form. This solves the problem of carryover-dependent NHEJ. They have also identified a germline promoter, from the zpg gene, that has minimal (though not completely absent) maternal carryover, limiting the fitness costs to heterozygotes. Work describing the use of this promoter (and the associated HEG) to bring about complete population elimination has been described in two recent papers, Kyrou et al., 2018, and Simoni et al., 2020.*

*What has not been reported is a careful description of how the authors characterized zpg-HEGs and those of earlier generations, which fared less well, exactly how non-optimal characteristics can lead to failure, and how (quantitatively) constructs that avoid these pitfalls do so. These details are critical as they inform the field about how drives can and will fail, and how to characterize them so as to understand where and when this will occur. Thus, while this manuscript comes after reports of dramatic success in bringing about suppression, it is a very important work in its own right. It provides a careful blueprint for understanding how a HEG should be characterized: how to "look under the hood" using genetics and sequencing to determine exactly what is going on with the parts that make up the HEG.*

*The strategy taken is interesting, and involves targeting a gene, nudel in Drosophila, required for female fertility. The authors had previously targeted this gene only to have resistant alleles arise rapidly (earlier publications). They target it again here precisely because it fails, and thus the rate of failure and how failure occurs can provide insight into how to avoid it (dsx, because it always works does not provide this opportunity). HEGs were constructed that target nudel, with Cas9 being driven by one of three different germline promoters, zpg, nos and exu. Data from the earlier published experiments using the vasa promoter served as a benchmark for improvements.*

*Overall the experiments are very clear and the data convincing. The use of marked chromosomes to follow the generation and fate of alleles over multiple generations is really a tour de force of population genetic characterization of an evolving system, particularly in a non-model system. The conclusions are clear and provide a clear roadmap for others in terms of how to characterize HEG behavior. This work will become and set the standard for all work going forward– which is a good thing.*

*I have a series of comments below, but all of these are questions or comments asking for further clarification of some discussion or figures.*

*1. One thing I am unclear on in Fig. 2CEGI is what the marks are in the top row of each plot. I am guessing these may be fecundity plots for wildtype females crossed to wildtype males, since then the statistical significance numbers make sense. This is suggested by the WT symbol on the far left, but, it is not indicated explicitly in the figure legend.

Also, throughout the figures, including Fig. 2, the type used is very thin and not bolded. it would help a lot if all type and male and female symbols were bolded and in some cases perhaps made larger. There is plenty of space in the figure. The same goes for the schematic presented in panel A. All parts could be larger and more clear. The authors might also consider expanding the schematic a bit to try and capture some of the problems (resistance alleles, carryover) that are the topic of the figure. it takes a bit of thinking to understand the significance of the fact that they are not just looking at progeny of heterozygotes, but progeny of heterozygotes that came from a specific sex. This would be a nice place to illustrate the things that go wrong and how they depend on grandparents. This has not really been presented schematically before, I think. Finally, panel A does not seem to be noted in the legend.*

**We have expanded the figure legend to describe panel A in more detail and have tried to convey more clearly that the scheme is designed to parse out the effects of maternal and paternal transmission of the gene drive allele. The WT fecundity rates in Fig 2C,E,G,I should also now be more clearly labelled since we have labelled the x-axes of these plots.**

*2. The paragraph lines 222-233 is a central part of the story. it would be useful to expand some of the single sentences into a few sentences to explain the results. For example, what is the thinking on the reduced fecundity of nos-expressing males. Is this likely to be male carryover, or is it just that perhaps the expression of Cas9 in males in the nos expression pattern is a bit toxic somehow to sperm? It would also be useful to unpack a little bit the key sentence that describes the zpg results, in terms of what it tells us.*

**The short answer is, we don’t know. We wrote “The reason for the observed lower fecundity in *nos-CRISPR^h^* males is unexplained, given that the target gene affects female fertility. Possible explanations might include some unknown feature of the exogenous nanos promoter that affects sperm quality or general reduced fitness resulting from the transgenesis procedure itself.”**

*3. Figure 3 describes a truly elegant experiment designed to look at the effects of parental nuclease deposition, by following the fate of an existing R1 allele when in trans to a wildtype allele, and in the presence of Cas9 provided by one parental gamete type or the other through deep sequencing. This experiment also allows them to look for the appearance of new alleles due to Cas9 deposition..

The left panel, which schematizes the experiment, could be made much bigger and more bold. Also the colors for the different alleles are a bit too similar. it would be useful to make them more different and have a legend in the figure itself that makes clear which is which.

In the legend in part A there is no discussion of the F3 generation.*

**We have both expanded the legend and changed the colours of the different alleles in order to make them more distinct. We have also ensured that all of our figures comply with the PACE guidelines to ensure that they are sufficiently visible for reproducibility.**

*4. In Figure 4 the authors look at the what happens when homing does not occur. Are new alleles created, and if so what are they? There are a couple of things I am unclear about. First, in the cross scheme they have GD/DL twice in the F2, at 47%. Should one of these be GD/WT? Also, they do not explain in the text or the legend the significance of the docking line. Is this just an insertion at the target site that eliminates the target site and provides a molecular difference easily scored by sequencing and/or visually? In short, it would be useful to explain the screen a bit more. The text is a bit telegraphic as to what is being tested and how. Since this is an online journal I do not think there are strict length limits that require minimal descriptions.

Finally, it is interesting that most of the alleles rated in the female germline are R1. Is there something to speculate on here? Is it just that this specific site tends to mutate in a way that creates a specific R1, or might something else be going on?*
**Yes, embarrassingly we left an extra GD/DL annotation on the figure. We had spotted this earlier but obviously a non-corrected version was uploaded. We have corrected it and have expanded the description of the docking line in the figure legend - the reviewer is correct in that it is both gene drive insensitive and contains a GFP-linked cassette for tracking. With regards to speculating on some of the reasons for different outcomes we included the following in the main text “The apparent differences in the behaviour of this gene drive between the male and female germlines may reflect differences in the timing of nuclease activity and chromosomal repair in the two sexes. It may also suggest that early repair events in the female germline, when only a few germline stem cells are present and indeed only one of 16 germline cells becomes an oocyte, leads to a clustering of repair events among the eventual gametes”**

*5. In Figure 5 the authors report on the behavior of the zpg drive targeting nudel. We know it is going to fail, but
the question is, given what has been demonstrated in the earlier figures, how much better will it behave, and what do the alleles that stop it look like.

First, it represents a lot of work to show when and what the resistance alleles look like, at many different time points. This figure stands alone in the gene drive field in this respect.

The authors discuss the egg counts in panel A, but don't refer back to a specific figure panel as showing this.
In panel B it would be useful to explicitly label which experiment is which since there are six panels with two Y axes labels (which is hard to see because they are so small).*

**We now cite panel A in the main text and agree with the reviewer that it should be apparent from the figure which experiment each panel refers to. We have modified this accordingly.**

*it is unclear from their discussion of panel B what the data in the two sets of Y axes panels tells us, because they re not referred to. It is not that clear from the discussion what we take away from the figures versus the discussion, which focuses mostly on the number of mutant alleles that arise and how these are lower with zpg.*

**We have added text in the main text to make what is being shown more explicit - "We recorded the relative proportion of wild type alleles and various r1 and r2 alleles among remaining non-drive alleles as the gene drive invaded the population over time (Figure 5b)."**

*6. I also have a question that arises from the authors discussion. In the beginning of this section they say that "For the zpg-CRISPRh constructs, which have minimal fitness costs on fecundity in carrier females (containing one drive allele and one wild type allele), resistance did not show obvious signs of selection"

and then later on in the same section they say "The very late onset of resistance - if it is to occur - is a feature, not immediately intuitive, of this type of gene drive whose goal is suppression and where heterozygous ‘carriers’ are intended to show minimal fitness costs. Selection for r1 alleles only really becomes strong when the gene drive is close to fixation - when most individuals have at least one copy of the gene drive the relative advantage of an r1 allele over remaining WT alleles (that have a high probability of being removed by homing of the gene drive"

I appreciate what they are trying to say in the second paragraph. A key feature of a suppression HEG is that so long as heterozygotes are mating with wildtypes no costs are incurred–for an IDEAL HEG. However, in the earlier data, Fig. 2, they show that zpg females suffer a 50% fecundity cost. While this is much lower than the costs associated with vasa, I think most people would still consider a 50% fecundity cost to be quite large. It would be useful to rethink the wording for the first statement, and link these costs to the second statement. While in ideal world this is how a HEG should behave, how does a 50% fecundity cost alter this calculation. Also, is it correct to say that resistance evolves late, or just that it manifests its effects late in the drive process, at least when only ideal HEG behavior is considered.*

**We have changed the sentence that references ‘minimal fitness costs’ to “For the *zpg-CRISPR^h^* constructs, which showed the biggest improvement in fecundity of carrier females (containing one drive allele and one wild type allele) compared to previous constructs.**

“*or just that it manifests its effects late in the drive process?*” **- yes, this is what we are trying to say, and is an alternative way of saying it. We don’t say ‘evolves late’ (as we agree that would be problematic as an expression) but we say “Selection for r1 alleles only really becomes strong when the gene drive is close to fixation”**

*7. In figure 6, panel lower left, it looks like the blue line goes to zero and then undergoes a dramatic increase. Can you explain?*
**The data in figure 6 are the same dataset that was included in figure 5 and in the supplementary tables submitted and for some reason this point transposed as a zero during the making of this graph for figure 6. The correct value (2,253 eggs) was included in both figure 5 and supplementary data table. This error in figure 6 has now been corrected.**

*Also, it looks like the data points are collected every other generation, 2-4-6. In this context the three tick marks between each set of numbers are a bit confusing and could be eliminated (since they cannot mean anything biological).*
**As the reviewer points out this was just superfluous tick marks on the axis generated by the software used to make the graphs. We have corrected the axis**

8. In the conclusions they say
"Indeed when the zpg promoter was used to control expression of a homing based gene drive at the doublesex locus we noticed a paternal effect contributing to reduced fecundity that was not observed here [8]" Can the authors expand for a sentence or two on how this is likely to occur: cas9 expression having effects on sperm fitness or paternal carryover? or something else. It would just be good to have a clear understanding of possible sources, since even some non-homing clvr-type drives proposed for suppression require germline only effects, and Cas9 is generally considered not to show paternal carryover (though most researchers do not look carefully for it).
**We added a sentence to this section: ‘Thus we cannot exclude the possibility that local cross-talk with other nearby transcriptional enhancers or promoters, either endogenous to the genome or as part of additional elements of any gene drive construct, may affect its expression characteristics.’**

////////////////////////
Very minor points.

*1. We tracked the generation and selection of resistant mutations the course of a gene

It looks like the authors missed a word, perhaps "over" the course"*

**done**
*2. onset of target site resistance. Our results show that regulation of its expression

Maybe just change the word it to nuclease, to make clear what is being referred to*

**done**

*3. developing a robust gene drive.

...for homing based suppression*
**done - “when developing a robust homing-based gene drive for population suppression”**

*4. throughout the text the authors use the standard error of the mean, rather than standard deviation. This is just my personal opinion, but I find SEM values completely useless. It actually hides the diversity of outcomes that would be apparent on simple visual inspection of the data (like in figure 2). I like SD because it actually tells you something about the distribution. But, its just a suggestion.*
**We understand the point but for consistency with previous articles we have used SEM and other reviewers expressed a preference for SEM in the past**

*5. In the methods zpg-crispr is listed twice at line 488*

**done**

*6. is this the docking line referred to in the text? If so it would be useful to expand a bit in the text as to the gfp-based method used here to identify these individuals.
"500 female zpg-CRISPRh drive heterozygotes (F0) were crossed to a line heterozygous for a GFP+ marked knock-out AGAP007280 allele and allowed to lay eggs. Upon egg hatching, L1 larvae were sorted using COPAS (complex object parametric analyser and sorter), as in Marois et al. (2012). The very rare fraction of progeny (F1) that inherited the GFP+ marked knockout but not the zpg-CRISPRh allele due to lack of homing..."*

**as above, we expanded the text to describe the nature of the docking line, and we now refer to it here as ‘docking line’**
*7. The authors refer to references 15 and 20 for data showing that zpg RNA is localized.
Line 123 " deposited nos and zpg mRNA concentrate at the germ plasm due to regulatory signals present on the untranslated regions, which also further restrict translation of maternal mRNAs to the germline [15, 20]." I have looked at these references and do not see any evidence that zpg mRNA is localized to germ plasm. Is this correct? it is of course for nanos, but I am not familiar with any localization data for zpg, particularly since it acts much earlier during oogenesis.*

**The Tazuke et al citation (Fig 5) makes reference to the germ plasm location of zpg mRNA.**

*8. In supplement figure 2 legend the authors state that " (A) Increasing the fertility of heterozygous zpg-CRISPRh females by up to 80% can accurately predict the experimental data, suggesting the fertility assays underestimate their true fecundity."*
 *Can they explain this a bit more and relate it to the data presented in Figure 2 on fecundity" What exactly do they mean here and how does it relate to Figure 2? More generally, should some or all of these observations be moved to the main text given that they seem to offer a set of discussion points (in the legend) that are distinct from those made in the text regarding how one should think about the data presented in the text. In short, it is confusing to present data in the text and then say in the supplement we get a better fit when we increase fecundity over what is obsrved, therefore the data in the text must not accurately reflect the real situation.*

**We have modified this text to “One possible explanation for this is the sensitivity of the model to the fertility of female *zpg-CRISPR^h^* heterozygotes and the extent to which fertility is restored by resistant R1 alleles (Supplementary Figure 2) – two parameters that are particularly difficult to measure precisely from our simple measurements of female fecundity and whose real values may be somewhat different in the inter-generational cage invasion experiment”**

*9. In supplementary Figure 4 it is not clear what the green highlights of specific bases mean. Are these sequence polymorphisms from the reference genome, or something else?*

**Yes, these are sequence polymorphisms from the reference genome and we have indicated as much in the legend. We should have stated that.**

Reviewer #2: *The manuscript "Regulating the expression of gene drives is key to increasing their invasive potential
and the mitigation of resistance" explores and discusses the role of different regulatory sequences on the potential for Cas9 gene drives to generate resistance alleles which would break said drives.
The authors start by outlining the known issues around generating functional gene drives, and the scenarios via which resistance may develop and be selected for. They then detail the development of three new gene drive strains targeting a single locus associated with female fertility, and compare this to their previously published gene drive targeting the same locus.
This is a very interesting topic, and builds nicely on the authors previous work. Overall this a very nicely written and designed set of experiments, with only a few minor comments.
See below my comments ordered by section.
Abstract: I found the reference to modifications to germline regulatory sequences slightly misleading. This work compared several endogenous regulatory sequences, but didn't start editing or modifying these sequences.*

**We have replaced ‘modifications’ to ‘alternative’**

L72 - this sentence doesn't make sense grammatically

**fixed**

*Results:
Data analysis - The use of M-W tests and KW tests is fine - though the authors could consider whether using a linear model would eliminate the small risk of inflated type 1 error rates from running mutliple comparisons. In addition - a G-test/Chi-square approach to rates of inheritance would allow the calculation of odds ratios between lines e.g. males inherit at X rate compare to females - and direct rather than indirect comparisons between lines.
I would also caution that the use of these nonparametric tests indicates that the data has a non-normal distribution, in which case the use of means and s.e. should be avoided and replaced with median and IQR instead.
It would also be good to see the full statistics included here e.g. test statistics and sample sizes, not just the p value*

**The full list of test statistics and sample sizes, as well all raw data points, are included in the supplementary data, with a breakdown figure by figure with all the relevant raw data and summary data collated beneath a copy of each figure. As are the median and IQR values. However, for reasons laid out to reviewer 1 we think that for comparison across the published literature in the field we would prefer to use mean values (while accepting the limitations that the reviewer points out).**

*In Figure 2 - I struggled to compare these back to the numbers listed in text - are these different replicates that have been put together for analysis, if so this needs to be made explicit. In addition details in the legends should make it clear what points, lines and error bars represent.*

**We have made improvements to figure 2 and the legend that hopefully improve its clarity and also included more explicit reference to relevant panels of the figure as they are introduced in the text.**

*L211 - It would have been nice to see egg laying data as well as larval output, to look at egg laying and viability*

**Figure 2 is already very busy and we feel the most important parameter is the amount of ‘viable’ progeny but all the egg laying data is included in the supplementary data and tabulated under a copy of the relevant figure.**

*L253 - Brown-Forsythe and Welche are two different types of ANOVA - so which one was used?*

**Ordinary one-way ANOVA with Dunnett's multiple comparisons test was used and text has been updated to reflect that**
*Figure 4 - refers to R2 as non-resistant mutations and should be corrected*

**fixed legend**

*L316 - elaborated on later in the discussion but worth emphasising - that efficient drives can be release at low numbers and be as effective (if not as quick) at reaching high frequency - is a powerful finding*

**we have added the following to that section “i.e. a release of low numbers of mosquitoes with the gene drive can be as effective (if not as quick) at reaching high frequency.”**

*L329 - the pooling method could be more explicit - rather than refer to another MS briefly outline sample types and numbers*

**The pooled amplicon sequencing is described in detail in the Methods - and the sample types and numbers are referred to in individual methods sections when this technique was employed e.g ‘cage population invasion experiments’, ‘deposition testing’, ‘estimation of meiotic end-joining’ etc.**

L362 - In the creation of resistant mutants there is not much discussion of the role of mosaicism in the vas2 results. Why do you think you get more r2 alleles? Is it because the increased rates of mosaicism mean you detect these in individuals that also have uncut functional alleles?

**The perdurance of these r2 alleles for several generations would suggest they were fully heterozygous or homozygous in individuals, leading us to believe that any signal from mutant alleles in the soma of mosaics was negligible.**

*L370 - You outline a readily testable idea - that these different alleles results are stochastic and not based on selection - even without performing the experiment, it would be nice to see a brief outline of how you would confirm that*

**We take the reviewers point, but we think our data point to that. One could deliberately test the relative restoration fecundity in assays with each of the different r1 alleles that we recovered but in a sense the power of our experiment is it shows the dynamics of generation of these alleles and how they fare under real ‘pressure’ of a population.**

*L374 - speculation on the idea that there are fewer r1 allele variants in the 'new drive' there could also be other explanations? Lakc of mosaicism in these lines means fewer alleles are able to restore fertility to 100%? Or that timing of ediitng plays a role in the rate of NHEJ, so fewer indels generated?*

**Here we are referring to the reduced complexity of the range of alleles within the NHEJ class, rather than the absolute number, which we have already shown is reduced with these constructs.**

*Reviewer #3: In this study, the authors assess the performance of several promoters for Anopheles gambiae Cas9-based gene drives. They find that the Zpg promoter has superior performance compared to the Nos and especially the Exu promoters in terms of drive inheritance. The Zpg promoter also outperforms the previously utilized Vas2 promoter by reducing somatic expression, thus restoring partial fertility to females with the gene drive. Overall, this is a useful paper that provides new insights into gene drive mechanisms. The study is technically sound, assuming that the comments raised below can be addressed.

Some of the results presented in this paper have already been described in a paper published by the same group in Nature Biotechnology in 2018. However, an earlier version of the current paper was available as a preprint (*[*https://www.biorxiv.org/content/10.1101/360339v1?versioned=true*](https://protect-eu.mimecast.com/s/YqUHC5Rk4CZRvyrHllZWJ?domain=biorxiv.org)*) at that time, and this preprint is cited in the Nature Biotechnology paper.

Specific comments:

1. Within each construct, did the individuals with lower fertility also have lower drive inheritance among their offspring? Exu seems to have a different horizontal axis scale than the other four panels of Figure 2, which the authors might want to adjust for ease of comparison.*

**Adjusted**

*Why is there no statistically significant difference between the Nos male drive inheritance between paternal and maternal drive alleles? Can this be double-checked? It looks like a fairly sizable difference.*

**We double-checked this and it doesn’t come out as significant.**

*2. Why was the wild-type fecundity different for each promoter, when these insects should have been free of the transgene? Were they part of the same batch as the transgenic mosquitoes of each promoter, or were they all independent sets of experiments? This should be noted. If the latter, this possibly indicates confounding batch effects when assessing the relative fecundity of the different transgenic lines. In this case, probably only the larger differences should be considered statistically significant, or an alternative analysis should be performed that takes batch effects into account using all wild-type crosses for each comparison.*

**Given variability in bloodmeal batch/insectary conditions etc we find it most robust to have a matched wild type control. We have noted this in the methods as per the reviewers suggestion**

*3. Why was male fecundity reduced for nos and exu (but not vasa and zpg) if the target gene only affects female fertility? Could this be a line effect (the line of transgenics simply being less healthy in some cases due to the transgenesis itself, rather than the specific transgenes) or a batch effect as described above?*

**The reason for the observed lower fecundity in *nos-CRISPR^h^* males is unexplained, given that the target gene affects female fertility. Possible explanations might include some unknown feature of the exogenous nanos promoter that affects sperm quality or general reduced fitness resulting from the transgenesis procedure itself.**

*4. Why was fecundity not determined for the vasa males with drive mothers? Why did these males have slightly biased inheritance toward the gene drive if they were heterozygous for a resistance allele? Also, “ND” should be defined in the legend.*

**Because these are not heterozygous for the resistance allele, but rather they are mosaic resulting from deposited nuclease and therefore a significant portion of germline does not have opportunity to show homing-based drive. This leads to a reduction in overall observed homing. We have added definition for ND in legend**

*5. The cage data seems to deviate from the model simulations, especially for eggs laid. This could be due to a model parameter mismatch, but a presumably more likely explanation would be that the effective population size in the cages was lower than the census size, yet the latter was used in the model. It would therefore be interesting to adjust the model to a lower population size if possible. This issue of effective population size should at least be noted in the results.*

**This section was mostly talking about gene drive trajectory but for eggs laid it is certainly true that effective number of females contributing to next generation will play a big part. We added the text “Alternatively, there may be extra sources of stochastic variation such as variation in distributions of egg number and degree of participation of females in mating not captured in the model.”**

*6. Lines 266-271: in this experiment, the authors did not specifically select individuals which did not develop resistance alleles in the embryo to test for shadow drive. Since resistance allele formation in the embryos was near total for vasa, any “signal” for shadow drive would be minimal. For the other promoters, lack of any resistance allele formation in the embryo indicates that there is little to no maternal deposition, which would also rule out the possibility of shadow drive (if no Cas9 at early stages, none would be available later in the germline). At best, the authors can probably rule out “extremely strong” shadow drive (note that the Drosophila studies found at most moderate shadow drive).*

**While this experiment was not looking for vasa-driven shadow drive of a gene drive allele it was designed with the sensitivity to pick up vasa-driven shadow drive of the r1 resistant allele since it was balanced against a wild type cleavable allele.**

*7. Lines 292-294: early germline resistance alleles were also seen in references 10, 13, and 32, which could be cited here as well.*
**Cited**

*8. The references on lines 423-424 might not be the most appropriate for showing that multiplexing improves the robustness of drives. Reference 21 didn’t involve using multiple gRNAs to improve the efficiency of a drive (the gRNA targeted other sites that were not involved in the main drive), and reference 37 was a different form of gene drive and thus not fully relevant to the question of multiplexed gRNAs in homing drives. Reference 12 would be good here, as would this new paper:*[*https://www.pnas.org/content/early/2020/09/11/2004373117*](https://protect-eu.mimecast.com/s/3TLeC6BlNHrMDR4s9W1rF?domain=pnas.org) *In line 425, reference 37 did successfully reduce r1 resistance alleles, possibly through targeting constrained sites, but the above PNAS reference would also be a suitable demonstration of this. Really, though, only reference 8 conclusively shows the importance of constrained target sites.*

**We have changed the text to make the wider point about feasibility of expressing multi-guides as part of drive constructs (of different flavours) in insects and I think the current references are applicable, I have also included the suggested reference which is very recent and is another example of a multi-guide construct**

.
*9. The authors should provide their Mathematica modeling code as a supplement or in a permanent online repository such as GitHub.*

**Currently, we have deposited the full mathematical formulae used in the model as well as tabulated sets of all parameter values used in the model. The modeller responsible is currently on extended leave. The code is part of a larger program with unpublished code so I cannot lead it to github at present.**

*10. A new paper has recently used the nos promoter in Anopheles and should probably be cited:* [*https://www.pnas.org/content/117/37/22805*](https://protect-eu.mimecast.com/s/swhVC7pm0iAyqrBCkaDg9?domain=pnas.org)

**I would prefer to add this as a note in proof**

**Have all data underlying the figures and results presented in the manuscript been provided?**
Large-scale datasets should be made available via a public repository as described in the *PLOS Genetics* [data availability policy](https://protect-eu.mimecast.com/s/LyaGC3ygXfpxBjrTve770?domain=journals.plos.org), and numerical data that underlies graphs or summary statistics should be provided in spreadsheet form as supporting information.

Reviewer #1: Yes

Reviewer #2: Yes

Reviewer #3: Yes

PLOS authors have the option to publish the peer review history of their article ([what does this mean?](https://protect-eu.mimecast.com/s/TQVvC8MnNi6Qrmys4xT5e?domain=journals.plos.org)). If published, this will include your full peer review and any attached files.


If you choose “no”, your identity will remain anonymous but your review may still be made public.


**Do you want your identity to be public for this peer review?** For information about this choice, including consent withdrawal, please see our [Privacy Policy](https://protect-eu.mimecast.com/s/JGnvC9DoNfkx1JwUB0pYq?domain=plos.org).

Reviewer #1: **Yes:** Bruce A. Hay

Reviewer #2: No

Reviewer #3: No
