# Supplementary material for: Regulating the expression of gene drives is key to increasing their invasive potential and the mitigation of resistance
Source: PLoS Genet. 2021 Jan 29;17(1):e1009321. doi: 10.1371/journal.pgen.1009321 (PMC7886172; doi:10.1371/journal.pgen.1009321)
Supplement: Attachment — Submitted filename: Response to Reviewers.docx [file pgen.1009321.s014.docx]

Dear Drs Edwards and Barsh,

Once again, we would like to thank the reviewers for the careful and considered review of our manuscript and welcome the fact that it has improved its clarity. Reviewers 1 and 2 were happy with all the corrections made while reviewer 3 still had some items requiring further clarification. We have answered each of these in line below and submitted an updated copy of the manuscript file that incorporates. I trust that with this response, and a very thorough and extended review process through two rounds, the manuscript will finally be given editorial approval and made available to the public, as we feel it contains important information for the field.

Yours sincerely,

Tony Nolan

**Reviews**

Reviewer #1: All of my concerns have been addressed adequately.

Reviewer #2: This is my second review of The manuscript "Regulating the expression of gene drives is key to increasing their invasive
potential and the mitigation of resistance" I am pleased to see that my original queries have all been addressed, and in my opinion satisfactory responses to the other reviewer comments as well.
This is a welcome new piece of research to the field of gene drive development and I will be happy to see it published

Reviewer #3: The authors have addressed some of my points, but there are still some remaining issues I feel should be addressed before publication.

**Response:**

1. Differences in inheritance between paternal and maternal alleles in the Nos male drive

“The authors note that they double checked and found that there are indeed no significant differences. This seems strange, so I ran a Mann-Whitney test myself. Indeed, it was not significant (p=0.1). However, the difference between the female lines (with either male or female drive parents) only had p=0.03 instead of p<0.001 as shown in the figure. I’d therefore suggest rechecking these statistics.”

We are happy that the full datasets that we have supplied allow a rigorous examination of the analyses applied and we welcome this. There is likely no discordance between the output of the Mann-Whitney test calculation itself but rather the interpolation to find the P-value, an issue that arises when there are lots of tied values. In our case the software we used (PRISM by Graphpad) to analyse the data, which uses a standard method to correct for ties when it computes the U, i.e. the sum of the ranks, but there is not a standard method to get a P value from these statistics when tied-ranks are present. Prism reports an exact P value when the data are less than 100 and it does so by “tabulating every possible way to shuffle the data into two groups of the sample size actually used and computes the fraction of those shuffled data sets where the difference between mean ranks was as large or larger than actually observed”. In this situation, Prism reports an exact P-value that is the upper endpoint of a range of possible P-values. We have added a note on this in the Methods to specify this. We have also re-checked all the P values using both methods for all crosses and there is no change on the bound of the P-value with the exception of the case mentioned, where both give significance, but at varying bounds. The cross in question has a particularly high number of tied values due to the 100% inheritance seen on many occasions.

2. and 3. Difference in wild-type fecundity for the different promoters:

“..some of the conclusions about fecundity being reduced in some lines should probably be softened in the results section, and the possibility of these batch effects noted there in the results, not just the methods.”

We have added the following sentence to the Results section:

“*We also note that the measure of fecundity in these experiments is marked by high inter-experimental variability and that, despite the inclusion of a wild type control every time, we cannot exclude the possibility that batch effects can contribute in part to some of the differences observed between lines*.”

4. Deviations between cage data and model simulations.

“I’m generally happy with how the authors revised this. However, I feel they should also mention that variation in male mating success has a large impact on the level of stochastic variation in the cages.”

We have modified the sentence to read: “*Alternatively, there may be extra sources of stochastic variation such as variation in distributions of egg number and degree of participation of females and males in mating not captured in the model.*”

5. Shadow drive.

“The authors state in their reply that “While this experiment was not looking for vasa-driven shadow drive of a gene drive allele it was designed with the sensitivity to pick up vasa-driven shadow drive of the r1 resistant allele since it was balanced against a wild type cleavable allele.”

Yes, I understand this. However, shouldn’t the r1 allele be able to do shadow drive only if a wild-type allele is available for conversion? It looks like most wild-type alleles were converted to resistance alleles in the early embryo in this experiment (only about 5% appear to have remained wild-type), hence the reduction in power because they would not be available for conversion in the germline. I thus don’t think the authors can generally rule out shadow drive at the level of the Drosophila studies, even though very high levels of shadow drive can indeed be ruled out.”

We have investigated three different promoters and their performance in controlling the expression of a gene drive construct. Two of these lead to very little deposition of nuclease that could feasibly produce shadow drive. The third promoter leads to large amounts of deposited nuclease activity that results in de novo end-joining mutations rather than gene conversion in the germline. Thus we did not detect shadow drive under 3 scenarios, including one using the ortholog of the promoter sequence that did produce this effect in *Drosophila*. We stand by these observations and our language is accurate on this – we state that “any contribution of deposited nuclease to homing of these alleles in the germline is at best minimal, at least in our experimental setup” – without claiming that the phenomenon cannot exist in the mosquito.
